# Field assessment of physiological and yield responses of potato to glyphosate hormesis

**DOI:** 10.1038/s41598-025-34882-8

**Published:** 2026-01-07

**Authors:** Abbas Ghafori, Hassan Karimmojeni, Shahram Torabian, Salar Farhangi-Abriz

**Affiliations:** 1 Department of Agronomy and Plant Breeding, College of Agriculture, Isfahan University of Technology, Isfahan, 84156-83111 Iran; 2 Agricultural Research Station, Virginia State University, Petersburg, VA USA; 3 Agricultural Research, Education and Extension Organization (AREEO), Cotton Research Institute of Iran, Gorgan, Iran

**Keywords:** Marketable yield, Dose–response, H_2_O_2_ concentration, Malondialdehyde content, Antioxidant enzyme activities, Biochemistry, Environmental sciences, Physiology, Plant sciences

## Abstract

Hormetic effects refer to the phenomenon in which lowdoses of certain herbicides, such as glyphosate, stimulate plant growth rather than inhibit it. A field experiment was conducted using six glyphosate application rates (5, 10, 20, 40, 80, and 160 g a.e. ha^−1^), along with an untreated control on potato plants. The activity of enzymatic antioxidants, including catalase (CAT), ascorbate peroxidase (APX), and guaiacol peroxidase (GPX), as well as levels of malondialdehyde (MDA), hydrogen peroxide (H₂O₂), and leaf protein content, were evaluated, along with plant height, tuber yield, and tuber size distribution. The highest yield was recorded at a low glyphosate dose of (20 g a.e. ha^−1^), where tuber production increased by approximately 30% compared to the control. This treatment also resulted in a 54% increase in marketable tubers (> 150 g) and a 14% increase in total biomass. In contrast, the highest glyphosate rate (160 g a.e. ha^−1^) significantly reduced total yield, marketable tubers, total biomass, and plant height. This treatment also led to the highest levels of APX and GPX activity, along with elevated concentrations of MDA and H_2_O_2_. However, the highest CAT enzyme activity was observed at 20 g a.e. ha^−1^ glyphosate rate. These findings suggest that low-dose glyphosate applications, up to 20 g a.e. ha^−1^, can enhance potato yield; however, rates exceeding this threshold may negatively affect yield.

## Introduction

The potato (*Solanum tuberosum* L.) is a major agricultural commodity, recognized as one of the four most important crops globally, alongside wheat, maize, and rice. Its extensive cultivation and substantial nutritional value highlight its critical role in global food security and human nutrition^1,2^. Because of its wide adaptability, well-characterized physiology, and sensitivity to herbicide-induced stress, potato is a suitable model crop for assessing subtle physiological and biochemical responses to agrochemicals, including hormetic effects.

Weed management is a cornerstone of successful potato cultivation, with herbicides playing a vital role due to their effectiveness in controlling weed populations^3^. Given the potato’s status as a major global crop, increasing attention is being directed toward the development of integrated weed management strategies. These approaches include the use of herbicides, combinations of different herbicides, and the addition of adjuvants to enhance their efficacy^4^. Moreover, intensive herbicide use raises concerns about environmental impact and plant stress responses. Beyond their conventional inhibitory functions, some herbicides, when applied at reduced doses, can induce hormetic effects, stimulating plant growth by activating key metabolic pathways^5^. Hormesis is characterized by a biphasic dose–response, in which high concentrations of a chemical agent inhibit growth, while low concentrations stimulate it^6^. Herbicides, depending on the dosage, can therefore trigger a dose-dependent response. Interestingly, at very low concentrations, they may even elicit hormetic effects that enhance plant resilience to stressful environments^7^.

Glyphosate has been shown hormetic responses in various plant species when used at sub-lethal concentrations. Such low doses can enhance photosynthetic activity, antioxidant defense, and overall plant growth^8,9^. Furthermore, when applied in very small amounts, herbicides can lead to the accumulation of shikimic acid and enhance plants’ photosynthesis^5,10,11^. At sub-lethal doses, herbicides have been shown to induce hormesis, promoting growth in various crops through mechanisms such as enhanced antioxidant defenses, improved nutrient uptake, and stress tolerance^11,12^. However, despite accumulating evidence in other crops, information on glyphosate-induced hormesis in potato remains limited. Application of reduced concentrations of glyphosate during the flowering stage is supported by evidence showing that it can show hormetic responses in plants, including enhanced antioxidant activity, photosynthesis, and growth stimulation^13^. Flowering is also a window during which the plant’s systemic response to stress is most pronounced, making it an optimal time for triggering beneficial low-dose herbicide responses. Prior studies have demonstrated that sub-lethal glyphosate doses applied at or near flowering can improve physiological efficiency and tuber initiation in potato and other crops by modulating stress-related metabolic pathways^13,14^. Nevertheless, the optimal dose range for inducing beneficial hormetic effects without causing toxicity remains unclear, representing a key scientific gap. Despite these potential benefits, a critical agronomic concern must be considered. This could reduce the long-term effectiveness of glyphosate-based weed management strategies and threaten the sustainability of integrated weed control programs*.* Thus, any application of glyphosate-induced hormesis should be approached with caution, ensuring that short-term productivity gains do not come at the expense of long-term agronomic resilience. Therefore, this study aimed to determine the physiological and biochemical responses of potato plants to different low doses of glyphosate, focusing on antioxidant enzyme activities, oxidative stress markers, and yield components. We hypothesized that sub-lethal glyphosate doses would enhance antioxidant enzyme activity and stimulate growth and tuber yield, whereas higher doses would induce oxidative stress and reduce productivity.

## Materials and methods

### Experimental conditions

This experiment was conducted between March and July 2023 at the research farm of Isfahan University of Technology, Isfahan, Iran (32.38°N, 51.39°E). The farm is situated at an altitude of 1,630 m and experiences a cool semi-arid climate with dry summers. The annual mean precipitation is 110 mm, and the average temperature is 14.5 °C. The soil has a clay loam texture with a field capacity of 23% and a permanent wilting point of 11.0%. It has a bulk density of 1.3 g cm^−3^, an electrical conductivity of 0.58 dS m^−1^, a pH of 7.5, and an average organic matter content of 1.07%. Also, a summary of the meteorological conditions recorded during the experiment is presented in Table 1. Potato was chosen as the experimental model because it is a globally important crop with well-defined growth and yield traits, and its sensitivity to oxidative stress makes it suitable for detecting glyphosate-induced hormetic responses. Disease-free potato seeds of the cultivar “Marphona”, possessing 2–3 eyes, were selected. After complete land preparation, hand shovels were used to make ridges for the treatments. Potato plants were planted at a density of 5.3 plants m^−2^ with a row spacing of 75 cm and an in-row plant spacing of approximately 25 cm on March 10, 2023. The area of each experimental plot was 6 m^2^ (3 × 2) and contained four ridges. No pesticide was applied and weeds were controlled manually. Plots were irrigated at 10- to 15-day intervals, with each irrigation delivering 6 ± 0.1 cm of water. Irrigation was applied at a depth of 6 cm, which was calculated based on soil moisture deficit and field capacity in the 0–30 cm root zone. This depth corresponds to the standard practice for potato cultivation in the region, ensuring adequate replenishment of soil moisture without waterlogging.

**Table 1:** Summary of meteorological conditions (rainfall, mean temperature, and solar radiation) during the experimental period (March–July 2023).

Parameter	Value (mean)	Notes
Rainfall (mm)	10	Low precipitation, typical of semi-arid climate
Mean temperature (°C)	21.3	Ranged from 12 °C (March) to 30 °C (July)
Solar radiation (MJ m⁻^2^ day⁻^1^)	22.5	High radiation during summer months
Relative humidity (%)	19	Declined progressively toward July

The present field experiment was conducted during a single growing season due to time and resource limitations. To enhance reliability, the trial was designed with adequate replications and randomized plots. Nevertheless, future studies should extend the assessment to multiple years to ensure reproducibility and repeatability of the results.

A dose–response experiment was conducted using six concentrations of glyphosate herbicide, along with a control, in a randomized complete block design with three replications. The glyphosate formulation used in this study was a 41% SL product manufactured by Monsanto, with a recommended field application rate of 1440 g a.e. ha⁻^1^ (equivalent to 3.5 L ha⁻^1^). In the dose–response experiment, treatments included 5, 10, 20, 40, 80, and 160 g a.e. ha⁻^1^, corresponding to approximately 0.35–11.11% of the recommended rate. These sub-field doses ranged from less than one three-hundredth to about one-ninth of the label rate. For precise application, the respective amounts were 0.3, 0.6, 1.2, 2.4, 4.8, and 9.6 mg a.e. per 6 m^2^ plot. To achieve the desired rate, appropriate volumes of the 41% SL glyphosate were calculated and diluted in water to prepare a spray solution with a total application volume of 330 mL per 6 m^2^ plot. This corresponds to a spray volume of 550 L ha^−1^. Glyphosate treatments were applied using a simple handheld compression sprayer at the flowering stage. The sprayer was manually pressurized before each application. During spraying, the nozzle was held approximately 50 cm above the crop canopy, and the operator maintained a steady walking pace to ensure uniform coverage. The volume of the solution was 330 mL for each test plot with an area of 6 m^2^. To avoid the effects of herbicide drift, a row of no-planting was maintained between the plots. During herbicide application, plastic sheets were placed around each treated plot to minimize drift. For biochemical and physiological measurements, three plants per plot were sampled. Plants were harvested when approximately 50% of the foliage had senesced. Fresh weight was recorded immediately after harvest, followed by oven-drying at 70 °C for 72 h to determine dry weight. Plant height was measured from the soil surface to the apex of the main stem.

### Measurement of antioxidant enzyme activities

The activity of antioxidant enzymes was evaluated one week after glyphosate application during the flowering phase. All measurements were conducted on the most recently fully developed, healthy leaves. Antioxidative enzymes were isolated from 100 mg of fresh leaf samples that were frozen in liquid nitrogen and stored at − 80 °C. Catalase activity was measured in a total volume of 3 mL of 50 mM Na phosphate buffer (pH 7.0), which contained 4.51 μL of H_2_O_2_ (30%) and 50 μL of enzyme extract (pH 7.8). Catalase (CAT, EC 1.11.1.6) activity was assessed by monitoring the breakdown of H_2_O_2_ over 2 min, focusing on the decrease in absorbance at 240 nm using a spectrophotometer (U-1900, Hitachi, Japan)^15^.

The activity of ascorbate peroxidase (APX, EC 1.11.1.11) was assessed at an absorbance of 290 nm for 1 min utilizing a spectrophotometer. One unit of APX was designated as the quantity of enzyme required to oxidize 1 μmol of ascorbate per minute^16^.

The activity of guaiacol peroxidase (GPX, EC 1.11.1.9) was measured in a total volume of 3 mL of 50 mM sodium phosphate buffer (pH 7.0), which included 4.51 μL of H_2_O_2_ (30%), 3.35 μL of guaiacol, and 50 μL of enzyme extract (pH 7.8). Peroxidase activity was assessed at 470 nm absorbance for 1 min using a spectrophotometer^17^.

The protein concentration was measured following the method outlined by Bradford^18^. The Bradford reagent comprised 50 mL of 95% ethanol, 100 mL of orthophosphoric acid, and 100 mg of Coomassie Brilliant Blue, with distilled water added to achieve a final volume of 1,000 mL. The protein content was evaluated using 3 mL of Bradford reagent mixed with 50 μL of protein extract, which was then incubated at room temperature for 30 min. Protein content was determined at 595 nm absorbance with a spectrophotometer, utilizing bovine serum albumin (BSA) as a standard^18^.

### Determination of H_2_O_2_ concentration and malondialdehyde content

The levels of hydrogen peroxide (H_2_O_2_) were assessed using the method outlined by Velikova et al.^19^. Leaf tissues (0.2 g) were blended with 2 mL of 0.1% (w/v) TCA. The homogenate was centrifuged at 10,000 × g for 10 min. Subsequently, 0.5 mL of the supernatant was combined with 0.5 mL of 10 mM potassium phosphate buffer (pH 7.0) and 1.0 mL of 1.0 M KI. The absorbance of the solution was measured at 390 nm. The concentration of H_2_O_2_ was determined using a standard curve.

The assessment of lipid peroxidation in the leaves was conducted by determining the malondialdehyde (MDA) levels, following the method described by Heath and Packer^20^, utilizing thiobarbituric acid (TBA). Leaves (0.2 g) were homogenized in 2 mL of 0.1% (w/v) TCA. Following centrifugation at 10,000 × g for 15 min, 0.5 mL of the supernatant was mixed with 1 mL of 0.5% (w/v) TBA in 20% TCA. The mixture was incubated at 95 °C for 30 min, then allowed to cool to room temperature and centrifuged at 10,000 × g for 15 min. The supernatant was analyzed with a spectrophotometer, and the absorbance was recorded at 532 nm. The value for non-specific absorption at 600 nm was subtracted from the 532 nm reading. The concentration of the MDA–TBA complex (red pigment) was calculated using the extinction coefficient of 155 mM^−1^ cm^−1^.

### Tuber yield and tuber size distribution

Tubers were harvested 100–110 days after planting. Yield was measured from a 2 m^2^ area within the two middle rows of each plot, while the two side rows served as buffer zones. Tubers were categorized into non-marketable (< 50 g), marketable category 1 (50–150 g), and marketable category 2 (> 150 g), expressed as a percentage of total yield. Defective tubers (green, rotten, damaged, cracked) were counted and used to calculate the defect rate^21^.

### Statistical analysis

Analysis of variance (ANOVA) was conducted using SAS statistical software (version 9.4), and a graph was created using Excel 2016 software. Treatment conditions were considered significantly different when F-test *P* values were ≤ 0.05, and means were compared using the LSD (least significant difference) test as appropriate.

## Results

The effects of glyphosate doses were significant for plant dry weight (Table 2) and for CAT and APX enzyme activities (Table 3). Moreover, glyphosate significantly influenced guaiacol peroxidase activity, MDA content, H_2_O_2_ concentration (Table 3), as well as plant height, yield, and its components (tubers < 50 g, 50–100 g, and > 150 g) (Table 2).

**Table 2:** ANOVA analysis of the effect of glyphosate on tuber yield, tuber weighing less than 50 g, tuber weighing between 50 and150 g, tuber weighing more than 150g, height(cm), and total biomass of potato.

Source of Variation	df	Total yield	Tubers < 50 g	Tubers 50–150 g	Tubers > 150 g	Height	Total biomass
Treatment	6	102,589**	463**	134**	183**	67.6**	93.5*
block	2	18816^ns^	0.43^ns^	5.28^ns^	6.27^ns^	0.90^ns^	23.8^ns^
Error	12	15,068	3.38	2.93	5.47	11.79	30.4

**significant at *p* ≤ 0.01; *significant at *p* ≤ 0.05; ns, not significant.

**Table 3:** ANOVA analysis of glyphosate effects on antioxidant enzymes, protein, MDA, and H₂O₂ in potato.

Source of Variation	df	CAT	APX	GPX	Protein
Treatment	6	0.126*	0.10*	0.15**	0.033^ns^
block	2	0.005^ns^	0.21**	0.002^ns^	0.024^ns^
Error	12	0.028	0.03	0.004	0.013

		MDA	H_2_O_2_
Treatment	6	161**	4999**
block	2	9.90*	154^ns^
Error	12	2.29	159

**significant at *p* ≤ 0.01; *significant at *p* ≤ 0.05; ns, not significant.

### Antioxidant enzyme activities and protein content

**Fig. 1:**
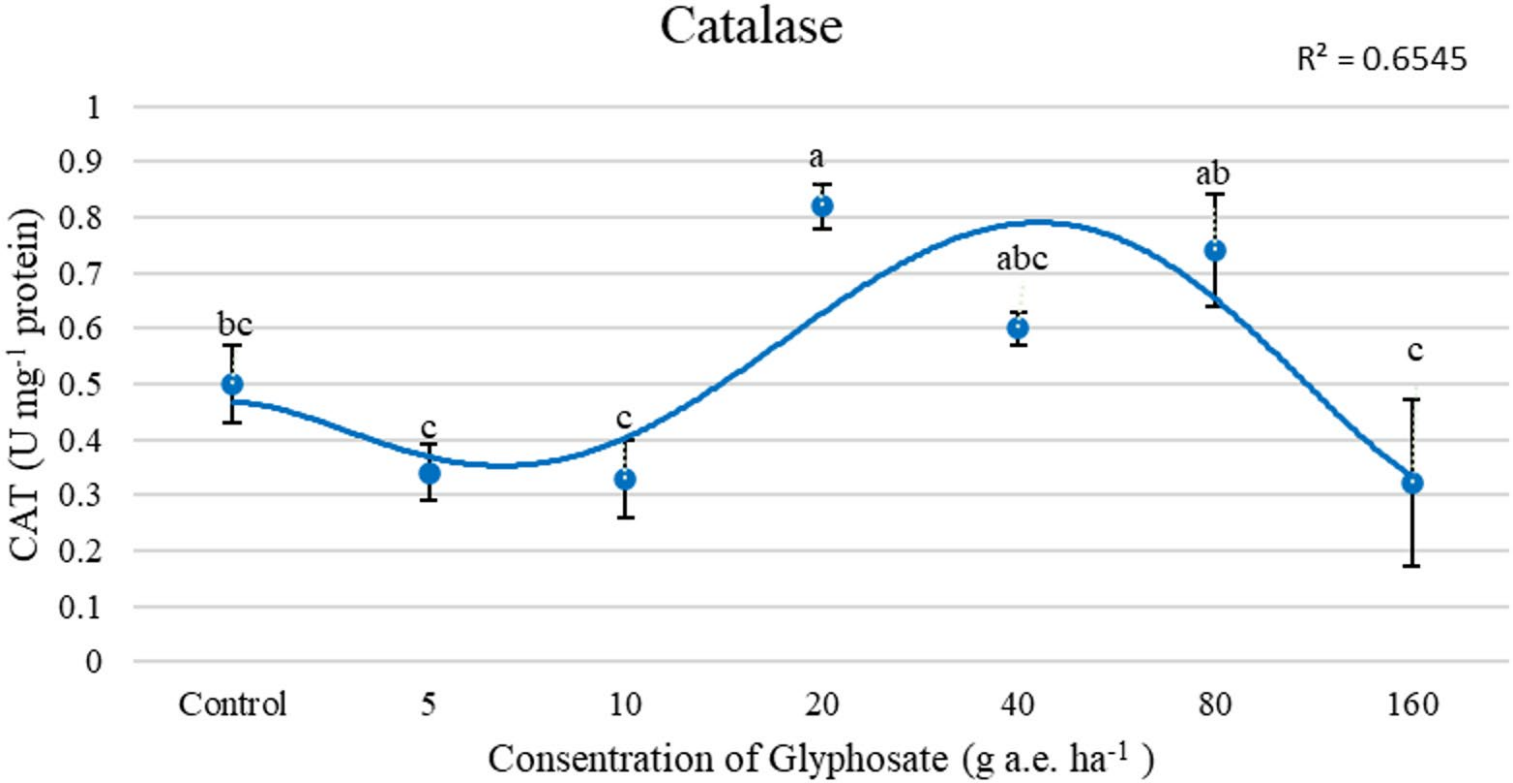
Effect of concentrations of glyphosate on the activity of CAT(U mg^-1^ protein). Bars with different letters indicate significant differences at *p* ≤ 0.05. Error bars are means ± standard error.

A significant increase in CAT activity was observed with the application of 20 g a.e. ha^−1^, exceeding 60% compared to the control, followed by a decreasing trend in CAT activity at higher doses (Fig. 1). APX activity, similar to GPX activity, increased with increasing herbicide rates (Table 4). The highest activities of APX and GPX reached 104% and 96%, respectively, with glyphosate application at 160 g a.e. ha^−1^ compared to the control. The lowest APX and GPX activities were observed in the control and 5 g a.e. ha^−1^ treatments. The activities of APX and GPX increased by 104% and 96%, respectively, when glyphosate was applied at 160 g a.e. ha^−1^, compared to the lowest rate of 5 g a.e. ha^−1^.

**Table 4:** Means of antioxidant activity APX and GPX of the potato plants under various glyphosate doses application.

Glyphosate dose	APX	GPX	Protein
g a.e. ha^-1^	U mg^-1^ protein		
Control	0.46±0.036^d^	0.16±0.012^d^	0.58±0.08
5	0.45±0.029^d^	0.17±0.010^d^	0.53±0.07
10	0.61±0.021^c^	0.35±0.013^c^	0.46±0.02
20	0.57±0.031^c^	0.39±0.011^bc^	0.43±0.04
40	0.87±0.034^b^	0.44±0.024^b^	0.41±0.12
80	0.84±0.022^ab^	0.40±0.034^bc^	0.45±0.04
160	0.92±0.045^a^	0.84±0.025^a^	0.47±0.06
LSD	0.11	0.06	0.20

Treatment means within each column sharing the same letters do not significantly differ according to the LSD test at *p* ≤ 0.05 (mean ± standard error). Ascorbate peroxidase (APX), guaiacol peroxidase activities (GPX).

### MDA content and H_2_O_2_ concentration

**Fig. 2:**
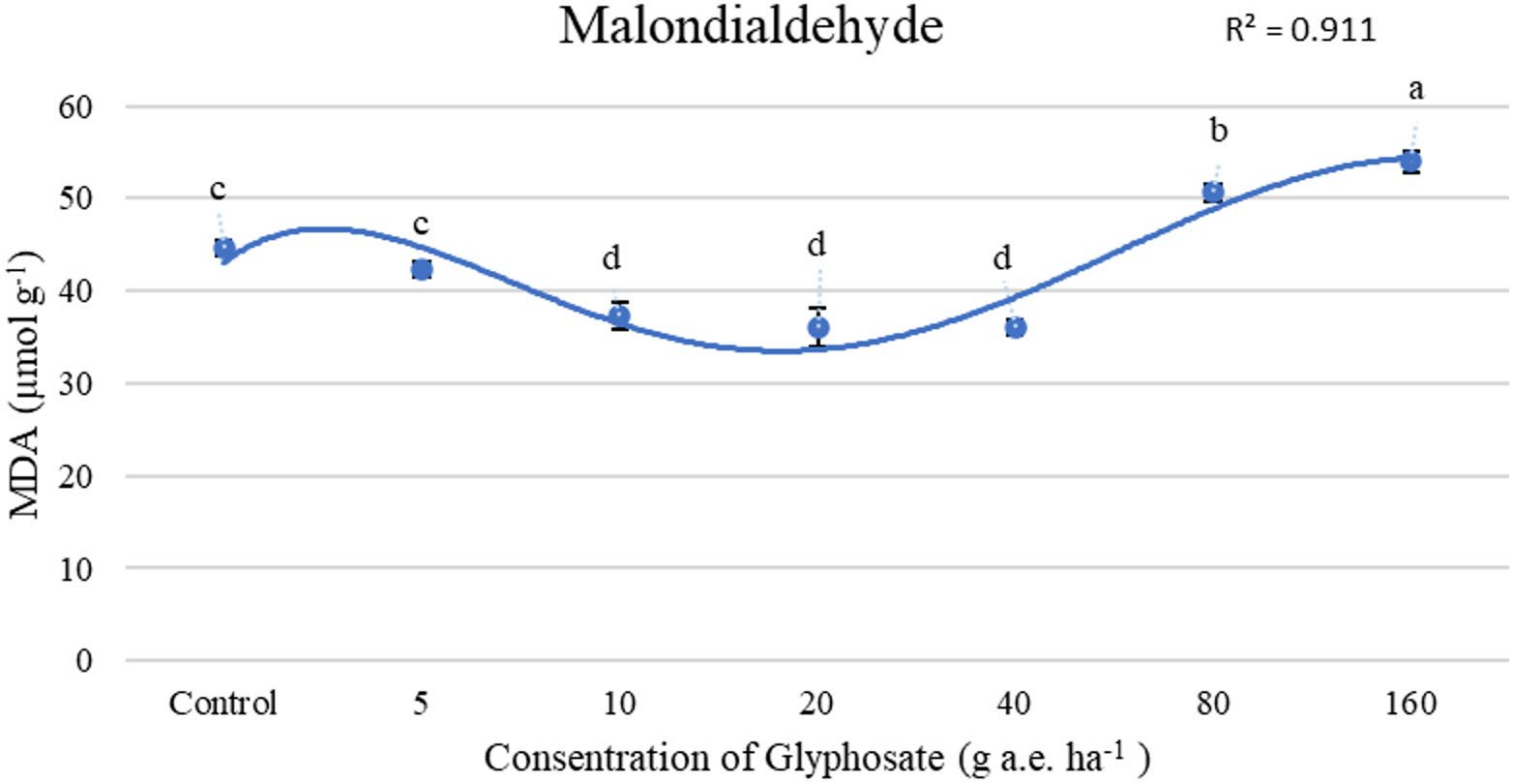
Effect of concentrations of glyphosate on MDA(µmol g^-1^). Bars with different letters indicate significant differences at *p* ≤ 0.05. Error bars are means ± standard error.

**Fig. 3:**
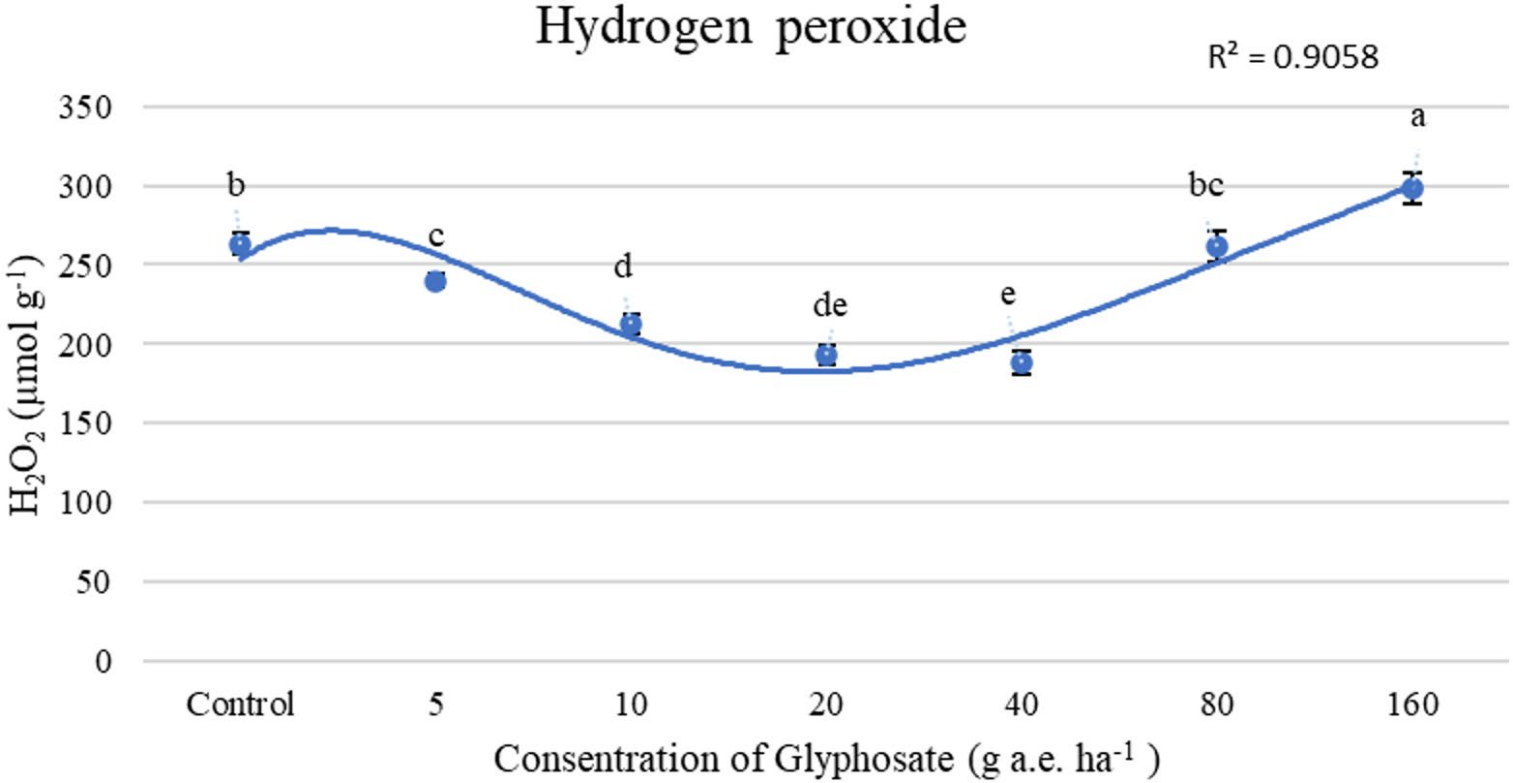
Effect of concentrations of glyphosate on H_2_O_2_(µmol g^-1^). Bars with different letters indicate significant differences at *p* ≤ 0.05. Error bars are means ± standard error.

Figures 2 and 3 presents the effects of glyphosate on oxidative stress markers, specifically, malondialdehyde (MDA) content and hydrogen peroxide (H₂O₂) concentration. The lowest MDA levels were observed in plants treated with glyphosate at 10, 20, and 40 g a.e. ha⁻^1^, as well as in the untreated control group. In contrast, the highest MDA content was found in plants exposed to the highest dose (160 g a.e. ha⁻^1^), exhibiting a 28.6% increase relative to the control, indicating increased oxidative damage (Fig. 2). Figure 3 shows that the lowest H₂O₂ concentration was observed with foliar application of glyphosate at 40g a.e. ha⁻^1^. However, excessive glyphosate exposure (160 g a.e. ha⁻^1^) led to a 13% increase in H₂O₂ concentration, further confirming the induction of oxidative stress at higher doses (Fig. 3).

### Growth traits

A decreasing trend in plant height was observed with increasing doses of glyphosate (Table 5). The difference in plant height was not significant at glyphosate concentrations of 10–80 g a.e. ha^−1^ compared to the control (Table 5). The application of 5 g a.e. ha^−1^ glyphosate significantly increased plant height (~ 17%) compared to the control. The hormetic effect of glyphosate on above-ground biomass was clear, as the highest plant dry weight occurred at a glyphosate application rate of 20 g a.e. ha^−1^. The lowest dry biomass was recorded at the glyphosate application rate of 160 g a.e. ha^−1^ (Table 5).

**Table 5:** Means of percentage tuber weighing 50–150 g total biomass, and plant height of the potato plants under various glyphosate dose applications.

Glyphosate dose	50–150 g	Total biomass	Plant height
g a.e. ha^-1^	%	g	Cm
Control	59.4 ± 2.1^a^	42.9 ± 0.10^ab^	47.3 ± 0.88^b^
5	56.1 ± 0.6^b^	36.9 ± 0.13^bc^	55.6 ± 0.33^a^
10	53.8 ± 0.2^bc^	46.0 ± 0.06^ab^	52.3 ± 0.88^ab^
20	51.2 ± 0.9^c^	48.9 ± 0.13^a^	49.6 ± 1.20^ab^
40	52.8 ± 3.2^c^	38.3 ± 0.14^bc^	52.6 ± 1.76^ab^
80	41.6 ± 8.1^d^	41.2 ± 0.09^abc^	48.0 ± 2.08^b^
160	42.4 ± 4.4^d^	32.6 ± 0.05^c^	41.0 ± 1.15^c^
LSD	3.04	9.80	6.10

Treatment means within each column sharing the same letters do not significantly differ according to the LSD test at *p* ≤ 0.05 (mean ± standard error).

### Yield and yield components

**Fig. 4:**
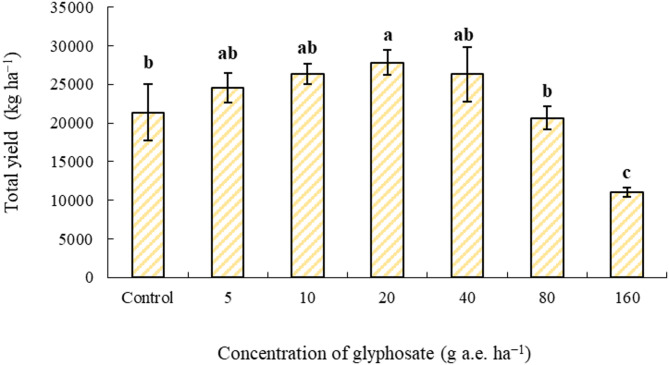
Effect of concentrations of glyphosate on total tuber yield (kg ha^−1^). Bars with different letters indicate significant differences at *p* ≤ 0.05. Error bars are means ± standard error.

The application of glyphosate at 5–40 g a.e. ha^−1^ induced a significant increase in the total tuber yield especially at 20 g a.e. ha^−1^, reaching approximately 30% above the control treatment (Fig. 4). This increase suggests a hormetic response, where low doses of glyphosate stimulate physiological and biochemical pathways that enhance growth and productivity. However, at a concentration of 160 g a.e. ha^−1^, tuber yield reduced by approximately 50% compared to the control (Fig. 4).

**Fig. 5:**
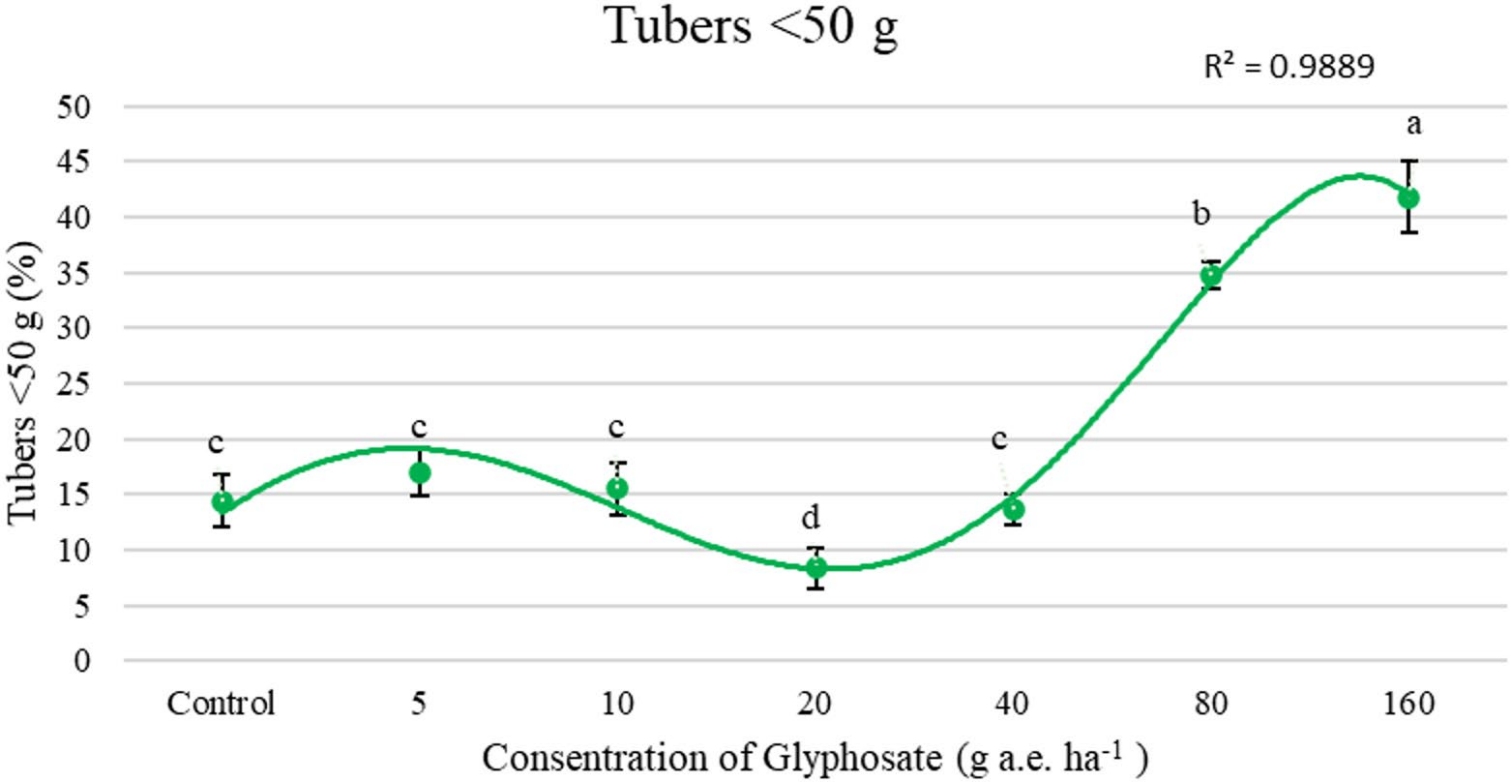
Effect of concentrations of glyphosate on percentage tuber weighing < 50 g (%). Bars with different letters indicate significant differences at *p* ≤ 0.05. Error bars are means ± standard error.

**Fig. 6:**
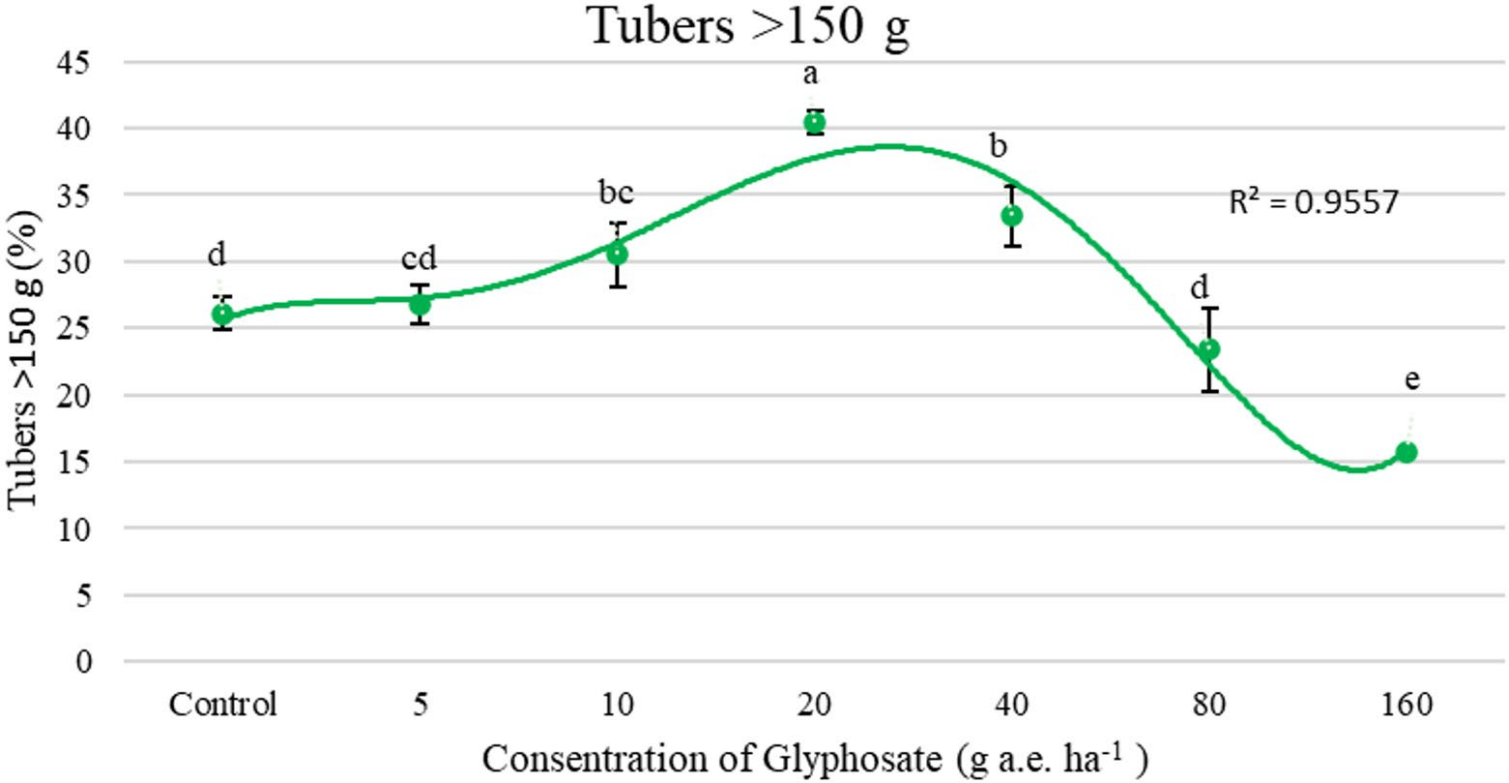
Effect of concentrations of glyphosate on percentage tuber weighing > 150 g (%). Bars with different letters indicate significant differences at *p* ≤ 0.05. Error bars are means ± standard error.

Figure 5 illustrates that as the glyphosate application rate increased, the proportion of smaller tubers (< 50 g) also increased. The highest percentage of tubers weighing less than 50 g was observed at 160 g a.e. ha^−1^. For tubers in the 50–150 g size category, untreated plants produced the highest yield, while the lowest percentages of these tubers were found at 80 and 160 g a.e. ha^−1^. In the case of larger tubers (> 150 g), the highest percentage was recorded at 20 g a.e. ha^−1^, showing a 54% increase compared to the control. These results indicate that when the glyphosate application rate exceeded 20 g a.e. ha^−1^, the percentage of tubers larger than 150 g decreased (Fig. 6).

### Correlation

Pearson correlation coefficients between total yield, tuber size distribution, plant height, biomass, antioxidant enzyme activities, protein content, MDA, and H_2_O_2_ levels are presented in Table 6. A positive and significant correlation was observed between total yield and the proportion of tubers > 150 g (r = 0.80), plant height (r = 0.66), and total biomass (r = 0.60). In contrast, total yield showed a strong negative correlation with MDA (r = –0.85) and H_2_O_2_ (r = –0.84). Additionally, a significant positive correlation was found between tubers > 150 g and plant height (r = 0.57), total biomass (r = 0.57). However, tubers > 150 g were negatively and significantly correlated with MDA (r = –0.88), while tubers < 50 g were positively and significantly correlated with MDA (r = –0.85). Plant height also showed negative correlations with MDA (r = –0.64) and H_2_O_2_ (r = –0.60). Furthermore, the proportion of tubers < 50 g was positively correlated with GPX activity (r = 0.55), MDA content (r = 0.88), and H_2_O_2_ (r = 0.75). A negative and significant correlation was observed between protein content and APX activity (r = –0.58) and GPX activity (r = –0.67) (Table 6).

**Table 6:** Pearson correlation coefficients between various traits in potato.

Coefficients	Total yield	Tubers < 50 g	Tubers 50–100 g	Tubers > 150 g	Plant height	Total biomass	CAT	APX	GPX	Protein	MDA	H_2_O_2_
Total yield	1											
Tuber < 50g	−0.80**	1										
Tuber 50–100 g	0.03^ns^	−0.25^ns^	1									
Tuber > 150 g	0.80**	−0.90**	−0.18^ns^	1								
Plant height	0.66**	−0.47*	−0.21^ns^	0.57**	1							
Total biomass	0.60**	−0.46*	−0.23^ns^	0.57**	0.17^ns^	1						
CAT	0.31^ns^	−0.25^ns^	−0.12^ns^	0.30^ns^	−0.02^ns^	0.36^ns^	1					
APX	−0.29^ns^	0.45*	0.05^ns^	−0.43*	−0.31^ns^	−0.13^ns^	−0.02^ns^	1				
GPX	−0.62**	0.55**	0.01^ns^	−0.56**	−0.63**	−0.42^ns^	−0.09^ns^	0.59**	1			
Protein	0.26^ns^	−0.37^ns^	−0.03^ns^	0.39^ns^	0.28^ns^	0.32^ns^	−0.08^ns^	−0.58**	−0.67**	1		
MDA	−0.85**	0.88**	−0.09^ns^	−0.85**	−0.64**	−0.45*	−0.15^ns^	0.37^ns^	0.54*	−0.40^ns^	1	
H_2_O_2_	−0.84**	0.75**	−0.02^ns^	−0.76**	−0.60**	−0.41^ns^	−0.34^ns^	0.27^ns^	0.3^ns^	−0.14^ns^	0.87**	1

**significant at *p* ≤ 0.01; *significant at *p* ≤ 0.05; ns, not significant.

## Discussion

### Antioxidant activity

Foliar application of glyphosate at 20 g a.e. ha⁻^1^ increased CAT activity. This suggests a hormetic response, where moderate doses (10–40 g a.e. ha⁻^1^) stimulate antioxidant defenses, but higher doses inhibit enzyme activity. In the study conducted by Ghafori et al.^22^, which investigated the hormetic effects of reduced glyphosate concentrations on niger plant (*Guizotia abyssinica*), it was found that CAT activity peaked at 6 g a.i. ha⁻^1^. This indicates that low dose of glyphosate exposure enhances the activity of antioxidant enzymes. Similar hormetic increases in CAT activity have been reported in other crops, indicating activation of antioxidant defenses against herbicide-induced oxidative stress^23^. The present results suggest that glyphosate at 10–40 g a.e. ha⁻^1^ induces a positive hormetic effect by mitigating ROS accumulation through activation of ROS-scavenging pathways. This effect could be attributed to the transient oxidative stress induced by glyphosate, which stimulates the plant’s defensive response and enhances antioxidant enzyme activities^24,25^.

Low doses of glyphosate may trigger these signaling pathways, leading to increased CAT activity, whereas higher doses could disrupt redox homeostasis and reduce enzymatic efficiency^26,27^. Though such molecular regulation remains hypothetical and was not directly assessed in this study. However, at excessive glyphosate concentrations, the overproduction of ROS may exceed the detoxification capacity of CAT and other antioxidant enzymes, resulting in oxidative damage and impaired enzymatic function^11^.

APX activity increased with increasing herbicide rates (Table 4). As a key enzyme in the ascorbate–glutathione (AsA-GSH) cycle, APX decomposes H_2_O_2_ into water and oxygen, thereby reducing oxidative damage^28^. The observed increase in APX activity indicates active detoxification of ROS in response to glyphosate-induced stress. APX, together with SOD and CAT, forms a key part of the antioxidant defense system under oxidative stress, with their activity regulated by stress-induced gene expression^23,29,30^. Both CAT and APX are key enzymes involved in H_2_O_2_ detoxification, although their roles vary depending on stress intensity, plant species, and tissue type^31^. While CAT primarily functions in peroxisomes to eliminate large quantities of H_2_O_2_, APX operates in specific cellular compartments, offering localized protection. The increased APX activity under higher glyphosate rates suggests activation of the AsA-GSH cycle to complement CAT and enhance the plant’s oxidative stress defense.

GPX activity increased with rising glyphosate concentrations (Table 4), suggesting its upregulation in response to herbicide-induced ROS production^25^. However, despite its elevated activity, GPX alone was insufficient to fully detoxify H_2_O_2_ at higher glyphosate levels. This may be due to ROS levels exceeding the enzyme’s capacity^32^ or GSH depletion under prolonged oxidative stress, which limits GPX efficiency^33^. High herbicide concentrations likely overwhelmed the plant’s antioxidant defense system, leading to excessive ROS accumulation, cellular damage, and lipid peroxidation, as reflected by elevated MDA levels (Fig. 2). The oxidative stress induced by herbicides is primarily attributed to an imbalance in ROS homeostasis^34^. Our results suggest that glyphosate at 10–40 g a.e. ha⁻^1^ may help protect plants by reducing H₂O₂ levels and mitigating damage from hydroxyl radicals during oxidative stress. This effect may be attributed to the enhanced activity of enzymatic antioxidants, which scavenge ROS and help maintain redox homeostasis^35^. In contrast, concentrations exceeding 160 g a.e. ha^−1^ triggered oxidative stress, highlighting a threshold beyond which glyphosate induces toxicity rather than hormesis. Mechanistically, the reduction in oxidative damage at 10 − 40 g a.e. ha^−1^ suggests that moderate glyphosate doses may act as mild stressors, activating signaling pathways that enhance stress tolerance and antioxidant enzyme expression. This hormetic response is in contrast to higher doses, where ROS overaccumulation surpasses the scavenging capacity of antioxidant systems, resulting in oxidative damage.

### Growth and biomass

Low glyphosate doses promoted vegetative growth, consistent with hormetic effects. The use of 5 g a.e. ha^−1^ glyphosate notably enhanced plant height when compared to the control (Table 5), supporting that mild oxidative stress can stimulate growth-promoting pathways. This finding aligns with Latorre et al.^36^, who reported that a sub-dose of 36 g a.i. ha^−1^ glyphosate promoted the growth and development of corn by enhancing plant height and leaf number. Similarly, Mobli et al.^9^ suggested that low doses of glyphosate might increase cell wall elasticity by inhibiting lignin biosynthesis, which can contribute to greater plant height. Moreover, Khan et al.^37^, demonstrated that tomato growth, photosynthetic activity, and chloroplast content improved with both foliar and soil applications of glyphosate at concentrations ranging from 0.03 to 1 mg L⁻^1^ in water or 0.03 to 1 mg kg⁻^1^ in soil. These findings support that sub-lethal doses of glyphosate can induce a hormetic effect^11^. The hormetic effect may stem from transient oxidative stress that activates antioxidant defenses, thereby enhancing cellular activity and growth^38^.

Glyphosate at 20 g a.e. ha⁻^1^ also enhanced above-ground dry weight (Table 5), reflecting improved carbon assimilation and nutrient use efficiency under mild stress conditions. This pattern aligns with the principle that low-dose stress can stimulate metabolic activity and growth, while excessive stress leads to inhibition^8^. Sub-lethal glyphosate doses have been shown to enhance plant growth, physiological efficiency, and enzymatic activity^22^. For instance, Nascentes^39^ observed increased leaf and stem dry weights in sugarcane under low glyphosate rates, likely due to improved carbon assimilation and nutrient allocation. Duke et al.^40^ also reported herbicide-induced increases in plant height, biomass, and protein content in various crops. These effects may result from mild oxidative stress that boosts antioxidant defenses and photosynthesis^41^. Glyphosate may also improve nitrogen-use efficiency and protein synthesis^42,43^, while promoting root development and nutrient uptake through changes in root architecture, ultimately supporting greater above-ground biomass^26,44,45^.

### Yield components

According to Fig. 4, total tuber yield increased with glyphosate application up to 20 g a.e. ha^−1^, where the highest yield was observed. However, further increases in glyphosate concentration led to a significant decline in yield, with the lowest yield recorded at 160 g a.e. ha^−1^. As glyphosate concentration increased, the proportion of nonmarketable tubers (< 50 g) also increased (Fig. 5). In the 160 g a.e. ha^−1^ treatment, 41% of the total tuber yield fell into the nonmarketable category, whereas the lowest proportion of nonmarketable tubers (8%) was recorded in the 20 g a.e. ha^−1^ treatment (Fig. 5). Moreover, glyphosate application at 20 g a.e. ha^−1^ significantly enhanced the proportion of marketable tubers (> 150 g), increasing their percentage by 40% compared to the control treatment (Fig. 6). These findings underscore the hormetic effect in improving tuber size and marketable yield, whereas excessive concentrations induce physiological stress and yield reduction. At glyphosate levels of 80 and 160 g a.e. ha^−1^, nonmarketable tubers accounted for more than 50% of the total yield, highlighting a threshold beyond which glyphosate toxicity negatively affects tuber development. De Moraes et al.^46^ reported that glyphosate application at 11.25 g a.e. ha^−1^ increased the dry weight of *Urochloa decumbens* by 30%. Similarly, Cesco et al.^47^ examined the hormetic influence of glyphosate on the vegetative and reproductive development of *Conyza sumatrensis* genotypes and found that doses below 11.25 g a.e. ha^−1^ enhanced leaf count, plant height, and dry biomass accumulation.

Overall, these findings indicate that optimizing glyphosate dosage within the intermediate range of 10–40 g a.e. ha⁻^1^ can enhance potato yield and quality by harnessing hormetic effects, while minimizing the risk of toxicity-related yield reductions.

### Study limitations

In this study, weeds were mechanically removed to eliminate confounding effects, allowing the experiment to focus solely on potato responses to sub-lethal glyphosate doses. While this approach clarified crop-specific outcomes, it is important to acknowledge that repeated exposure of weed populations to low glyphosate doses may impose selection pressure, potentially driving resistance evolution, altering susceptibility, or enhancing reproductive and dispersal capacities. Future research should therefore examine the ecological implications of glyphosate-induced hormesis in weed communities and assess its long-term contribution to herbicide resistance.

A further limitation of this study is the absence of photosynthetic and chlorophyll-related measurements, which could have clarified whether the observed growth and yield stimulation were associated with enhanced photosynthetic efficiency. Moreover, the proposed molecular and hormonal mechanisms remain speculative, as they were not experimentally validated. Incorporating photosynthetic, hormonal, and gene-expression analyses in future work would provide stronger insights into the physiological basis of glyphosate-induced hormesis. Also, future studies should include residue analysis to provide a more complete evaluation of food safety.

## Conclusion

This study highlights the potential of glyphosate-induced hormesis in enhancing potato productivity. A low dose of 20 g a.e. ha⁻^1^ significantly improved plant growth, tuber yield, marketable tuber weight, and total biomass, while reducing the proportion of nonmarketable tubers (< 50 g). In contrast, higher doses (160 g a.e. ha⁻^1^) induced oxidative stress, as indicated by elevated MDA and H₂O₂ levels, and suppressed both growth and yield. These results suggest that carefully managed, low glyphosate doses could have agronomic value for stimulating crop performance under certain conditions. However, such applications must be approached cautiously due to potential ecological risks, including unintended impacts on soil microbiota, non-target vegetation, and the possible acceleration of weed resistance. Further research is necessary to evaluate the long-term impacts of low-dose glyphosate application, particularly its effects on the nutritional quality of potato tubers, including starch, sugar, and carbohydrate content.

## Data Availability

The datasets generated during the current study are available from the corresponding author on reasonable request
